# Optimal timing of oral anticoagulation initiation in patients with acute ischaemic stroke and atrial fibrillation: a comprehensive meta-analysis and systematic review

**DOI:** 10.1136/openhrt-2024-003002

**Published:** 2024-11-27

**Authors:** Aravind Dilli Babu, Sahib Singh, Asher Gorantla, Mirza Faris Ali Baig, Ram Bhutani, Harika Davuluri, Lekshminarayan Raghavakurup, Bengt Herweg

**Affiliations:** 1 Internal Medicine, Sinai Hospital (LifeBridge Health), Baltimore, Maryland, USA; 2 Cardiology, SUNY Downstate Medical Center, New York City, New York, USA; 3 Internal Medicine, Asante Three Rivers Medical Center, Grants Pass, Oregon, USA; 4 Electrophysiology, University of South Florida, Tampa, Florida, USA

**Keywords:** Atrial Fibrillation, Electrophysiology, Stroke

## Abstract

The optimal timing for initiating direct oral anticoagulants (DOACs) for secondary stroke prevention in patients with atrial fibrillation and acute ischaemic stroke remains controversial due to concerns about haemorrhagic transformation. This study aimed to analyse the efficacy and safety of early versus late DOAC initiation. Following the Preferred Reporting Items for Systematic Reviews and Meta-Analyses guidelines, a systematic review was conducted, searching major databases (PubMed, Embase, Cochrane Library and ClinicalTrials.gov) up to May 2024. A total of 11 studies were identified, comprising nine cohort studies (75.5% weight) and two randomised controlled trials (RCTs) (24.5% weight), involving 13 020 participants. The early DOAC group (mean initiation 3.5±1.29 days) included 6250 participants, while the late group (5.7±1.25 days) had 6770 participants. Outcome measures included recurrent ischaemic stroke (RIS), intracranial haemorrhage (ICH), systemic embolism, major haemorrhage (MH), non-major haemorrhage (NMH) and all-cause mortality. Statistical analysis using the Cochrane Review Manager calculated ORs and 95% CIs via the Mantel-Haenszel random effects model. This pooled meta-analysis revealed that the early DOAC group had lower rates of RIS (2.2% vs 2.9%, OR 0.72, 95% CI 0.52 to 0.98, p=0.04, I^2^=40%) and ICH (0.51% vs 0.93%, OR 0.45, 95% CI 0.29 to 0.70, p<0.05, I^2^=0%) compared with the late DOAC group. Subgroup analysis of RCTs and cohort studies showed reduced RIS and ICH risks in the early DOAC group, with moderate heterogeneity. In the sensitivity analysis, the early group (<4 days) had a lower risk of RIS compared with the late group (>4 days) without a statistically significant impact on ICH. No significant differences in MH, NMH, systemic embolism or all-cause mortality were observed between either group; however, a limited number of RCTs and moderate heterogeneity weakened the conclusions. Additional RCTs are needed to provide more definitive insights.

## Introduction

 Atrial fibrillation (AF)-related acute ischaemic strokes (AIS) are frequently disabling or fatal.^1^ AF is associated with approximately one-third of all stroke events and a fivefold increased risk of stroke.^2^ AF is linked to a heightened risk of early AIS recurrence (up to 1.3% per day),^3^ particularly within 48 hours to 2 weeks.^4^ The risk of haemorrhagic transformation (HT), a potential complication of AIS, often occurs within the first few days after stroke onset is high, especially in the initial days following large cardioembolic stroke lesions and acute reperfusion therapy.^4 5^ Symptomatic intracranial haemorrhage (ICH) is linked to higher rates of mortality and morbidity and has been reported in 2–20% of patients.^5^ Oral anticoagulation (OAC) therapy decreases AIS in patients with AF by 64% and decreases all-cause mortality by 24%.^3 6^ However, OAC treatment is initially withheld after admission to reduce the risk of HT. ^7^ Despite evidence that early initiation of OAC may mitigate the risk of AIS recurrence,^8 9^ there are concerns regarding the increased risk of haemorrhagic complications,^10 11^ especially in studies that predominantly used vitamin K antagonist (VKA) or heparin drugs.^12^ However, the newer class of medications, such as direct-acting oral anticoagulation (DOAC), has changed this landscape.^8 9^

The lack of robust evidence supporting early OAC over late OAC,^13^ especially in studies from the pre-DOAC era,^4 12^ has caused variations in current guideline recommendations regarding the timing of anticoagulation initiation.^14 15^ The objective of this meta-analysis is to explore the effectiveness and safety of early versus late DOAC initiation for secondary stroke prevention in patients with AF and AIS.

## Methods

**Figure 1:**
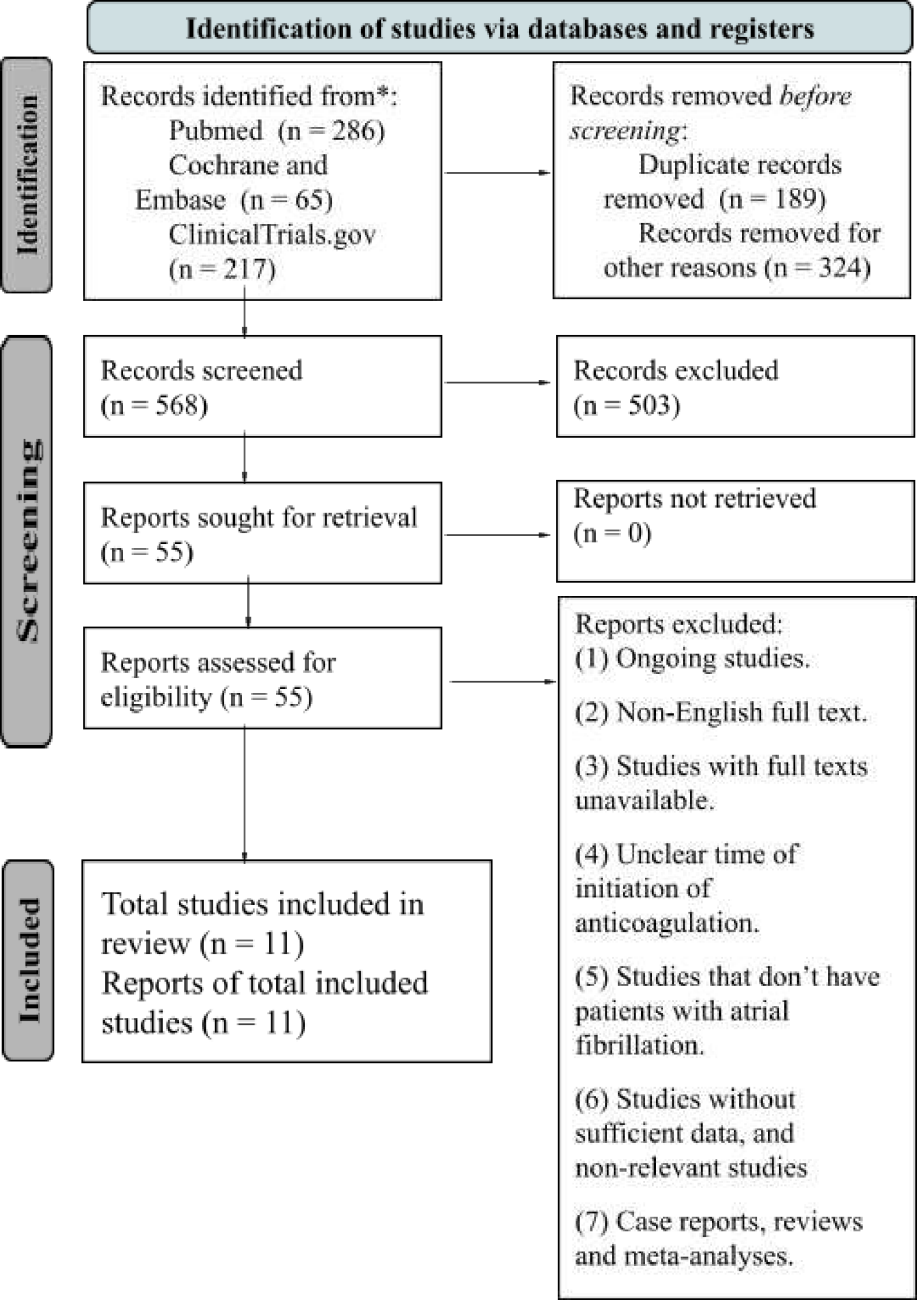
Preferred Reporting Items for Systematic Reviews and Meta-Analyses (PRISMA) 2020 flow diagram illustrating the search strategy. From Page *et al*.^41^

We adhered to Preferred Reporting Items for Systematic Reviews and Meta-Analyses (PRISMA) guidelines for this meta-analysis, focusing on our PICO question: Population (P)=adults (≥18 years) with AF and AIS; Intervention (I)=early or late initiation of OAC therapy; Comparison (C)=early versus late DOAC initiation; Outcomes (O)=clinical outcomes. Institutional review board approval was not needed as no individual patient-level data were used. Figure 1 presents the PRISMA 2020 flow diagram of our search strategy.

We conducted a systematic search of PubMed, Cochrane, Embase and ClinicalTrials.gov from inception to May 2024 using the keywords ‘Early’, ‘Late’, ‘Anticoagulation’, ‘Atrial fibrillation’ and ‘Stroke’. Inclusion criteria included randomised controlled trials (RCTs) or observational studies involving patients aged ≥18 years with AIS and AF. Additionally, studies that provided data for calculating the weighted mean difference and OR and reported at least one clinical outcome—recurrent ischaemic stroke (RIS) or symptomatic ICH—were included.

Exclusion criteria encompassed (1) ongoing studies, (2) non-English full-text articles, (3) studies with unavailable full texts, (4) studies lacking clarity on the timing of anticoagulation initiation, (5) studies lacking patients with AF, (6) studies lacking sufficient data, and non-relevant studies, including (7) case reports, reviews and meta-analyses. The search was independently performed by two researchers (ADB and RB), with conflicts resolved by a third researcher (SS). Initial screening was based on titles and abstracts, followed by a comprehensive review of the full articles to confirm relevance. Selected trials met the predefined criteria.

A total of 568 studies were initially identified across all databases. After applying exclusion criteria, 324 studies were eliminated, and 189 duplicates were removed. The remaining 55 studies were screened using PICO questions, leading to the inclusion of 11 studies (two RCTs and nine cohort studies) published between 2016 and 2023.^8^,^1016^ The included studies had varying timelines for DOAC initiation: 2–7 days for the early group (mean 3.5±1.29 days) and 5–14 days for the late group (mean 5.7±1.36 days).

Analysis was conducted using RevMan (V.5.4, The Cochrane Collaboration, 2020).^30^ The Mantel-Haenszel random effects model compared dichotomous outcomes (early vs late DOAC) across studies, calculating pooled ORs, 95% CIs, study weights and heterogeneity. A significance level of p≤0.05 was used, with an I² value >50% indicating substantial heterogeneity. Forest plots were generated for the final analysis, as illustrated in the PRISMA flow diagram (figure 1). The quality of observational studies was assessed using the Newcastle-Ottawa Scale,^31^ with scores of 7–9 considered ‘high quality’, while the Cochrane risk-of-bias tool was used for RCTs (online supplemental figure 2).^32^ Quality assessments were conducted independently by two review authors, resolving disagreements through discussion.

## Results

A total of 13 020 patients, with 6250 in the early DOAC group and 6770 in the late DOAC group, were included in this study. In the early group, 54.1% had a history of hypertension (3384 patients), compared with 52.2% in the late group (3534 patients). Similarly, 14% (917 patients) of the early group had diabetes, compared with 15% in the late anticoagulation group (1016 patients). Risk factors like hypertension, diabetes, prior stroke and AF were evenly distributed between the early and late OAC groups (table 1).

**Table 1:** Baseline characteristics of the patients

Study	OAC group	Patients (n)	Age (median)	Female	HTN	DM	Prior stroke	Prior atrial fibrillation	CHADS2-VASc score on admission	NIHSS score	HAS-BLED score	Intravenous thrombolysis	Thrombectomy
De Marchis *et al*^18^	Early AC	1362	78	637	1026	298	–	–	5 (4–6)	4 (1–8)	3.0 (2–3)	324 (24.0)	43 (3.6)
	Late AC	1188	77	567	899	275	–	–	6 (3–12)	6 (3–12)	3.0 (2–4)	281 (23.8)	53 (4.7)
Fischer *et al*^8^	Early AC	984	77	459	690	185	128	984	5 (4–6)	5 (2–12)	–	391 (39.7)	377 (38.2)
	Late AC	991	78	456	673	161	140	991	5 (4–6)	5 (2–11)	–	207 (21.0)	232 (23.5)
Kimura *et al*^25^	Early AC	785	77	314	532	125	148	785	3 (2–4)	8 (2–16)	2 (1–2)	230 (29)	135 (17)
	Late AC	1012	78	416	680	184	187	1012	3 (2–4)	6 (2–15)	2 (1-3)	218 (22)	99 (10)
Macha *et al*^24^	Early AC	239	78	114	214	76	–	–	–	4 (2–9)	–	–	–
	Late AC	4	68.5	3	3	0	–	–	–	15 (9–16)	–	–	–
Oldgren *et al*^9^	Early AC	450	78.4	207	333	81	79	450	–	4 (2–9)	–	132 (29.3)	65 (14.4)
	Late AC	438	78.3	203	338	91	76	437	–	4 (2–8)	–	65 (14.4)	56 (12.8)
Sharobeam *et al*^21^	Early AC	107	74.2	41	70	17	14	107	–	4 (1–8.5)	–	16 (17)	15 (16)
	Late AC	101	74.2	38	64	22	11	101	–	6 (2–13.5)	–	18 (18)	11 (11)
Wilson *et al*^20^	Early AC	358	75	147	211	54	77	358	5 (4–6)	2 (1–4)	–	43 (12)	–
	Late AC	997	76	433	635	168	200	997	4 (4–6)	6 (3–11)	–	220 (22)	–
Paciaroni *et al*^22^	Early AC	1061	–	–	–	–	–	–	–	–	–	–	–
	Late AC	992	–	–	–	–	–	–	–	–	–	–	–
Mizoguchi *et al*^23^	Early AC	223	74	78	150	69	43	140	5 (4–6)	3 (1–8)	3 (2–4)	50 (22.4)	12 (5.4)
	Late AC	276	75	101	203	107	61	146	5 (4–6)	5 (2–13.5)	3 (3–4)	62 (22.5)	20 (7.3)
Cappellari *et al*^17^	Early AC	97	78.8	60	78	12	16 (16.5)	76	5.3 (1.4)	7.8 (6.3)	2.9 (0.9)	50 (52)	–
	Late AC	50	79.3	31	39	8	10 (20)	38	5.3 (1.7)	8.8 (7.1)	3 (0.7)	21 (42)	–
Yasaka *et al*^28^	Early AC	584	77.1	–	–	–	–	–	2 (1–2)	8 (3–17)	2 (1–2)	424 (32.4)	
	Late AC	721	77.1	–	–	–	–	–	2 (1–2)	8 (3–17)	2 (1–2)	424 (32.4)	

ACanticoagulationDMdiabetes mellitusHTNhypertensionNIHSSNational Institutes of Health Stroke ScaleOACoral anticoagulation

The mean National Institutes of Health Stroke Scale (NIHSS) scores were 6.6 (SD±1.85) in the early OAC (DOAC) group and compared with 8.4 (SD±2.25) in the late DOAC groups. Similarly, the mean CHA2DS2-VASc scores were 4.3 (SD±0.96) in the early DOAC group compared with 4.3 (SD±1.1) in the late DOAC group. Additionally, the mean HAS-BLED scores were 2.48 (SD±0.34) in the early DOAC group and compared with the mean of 2.55 (SD±0.34) in the late DOAC group. The median age of the overall study population was 78 years, with females comprising 33%. The mean follow-up duration was 128.7±SD 69.4 days (table 2).

**Table 2:** Baseline characteristics of the studies

Study	Study design	Total included patients	Early OAC group (days)	Late OAC group (days)	Diagnostic studies	Newcastle–Ottawa Scale	Follow-up duration
De Marchis *et al*^18^	Prospective observational study	2550	<5	>5	MRI	7	30 days
Fischer *et al*^8^	Randomised controlled trial	1975	2–7	3–7	CT/MRI	8	90 days
Kimura *et al*^25^	Prospective observational study	1797	<4	4–10	MRI	7	90 days
Macha *et al*^24^	Retrospective observational study	243	<5	>7	CT/MRI	7	30 days
Oldgren *et al*^9^	Randomised controlled trial	888	<4	5–10	CT/MRI	7	90 days
Sharobeam *et al*^21^	Prospective observational study	208	<4	>4	MRI	8	30 days
Wilson *et al*^20^	Prospective observational study	1355	<4	>5	MRI	8	90 days
Paciaroni *et al*^22^	Prospective observational study	2053	<2	>3	CT/MRI	6	90 days
Mizoguchi *et al*^23^	Retrospective observational study	499	≤3	≥4	CT/MRI	8	2 years
Cappellari *et al*^17^	Prospective observational study	147	≤3	≥4	CT	8	7 days
Yasaka *et al*^28^	Prospective observational study	1309	≤3	≥4	CT/MRI	7	90 days

OACoral anticoagulation

**Figure 2:**
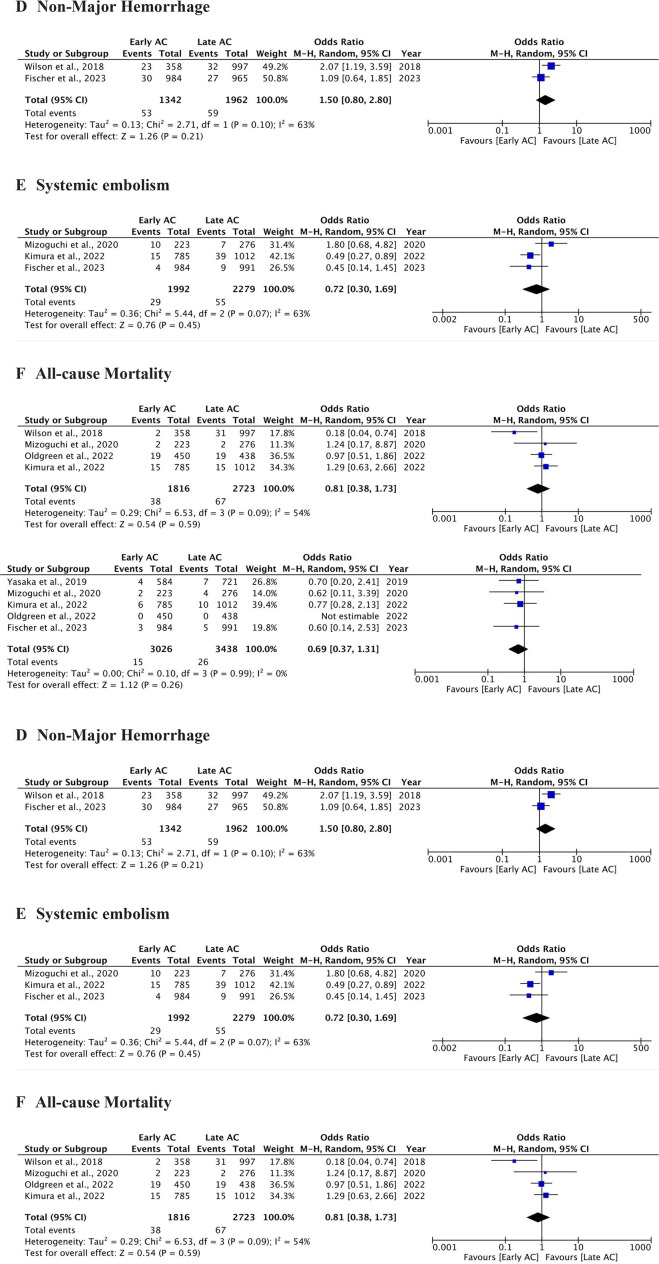
Forest plot showing differences between early direct oral anticoagulant (DOAC) and late DOAC groups. (**A**) Recurrent ischaemic stroke. (**B**) Intracerebral haemorrhage. (**C**) Major haemorrhage. (**D**) Non-major haemorrhage. (**E**) Systemic embolism. (**F**) All-cause mortality. AC, anticoagulation.

**Figure 3:**
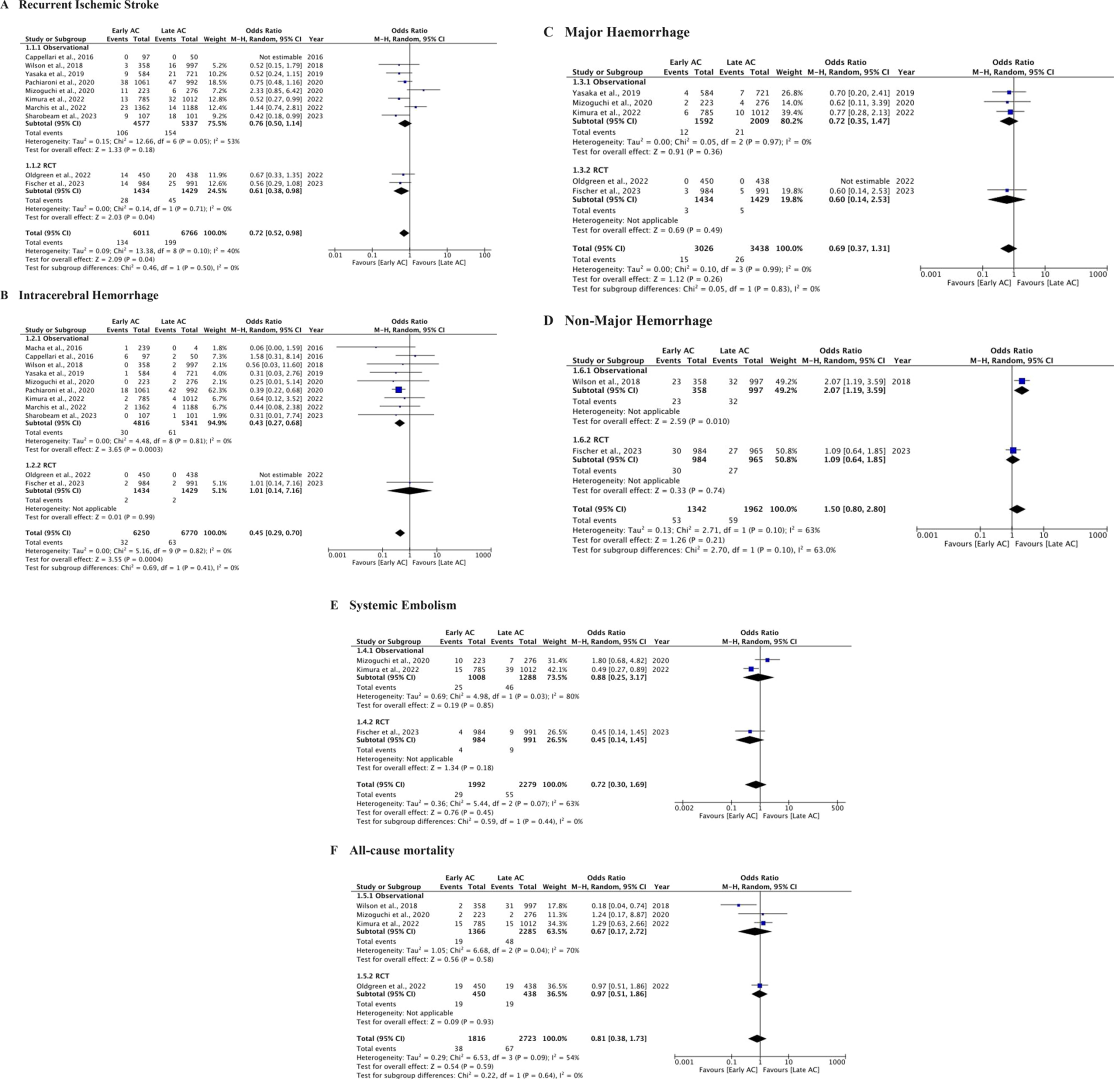
Forest plot showing differences among observational and randomised controlled trial (RCT) studies. (**A**) Recurrent ischaemic stroke. (**B**) Intracerebral haemorrhage. (**C**) Major haemorrhage. (**D**) Non-major haemorrhage. (**E**) Systemic embolism. (**F**) All-cause mortality. AC, anticoagulation.

In 10 studies,^89 17 18 21^,^23 25 28^ the outcome of RIS was assessed. In the early DOAC group (n=6011), there were 134 events (2.2%), compared with 199 events (2.9%) in the late DOAC group (n=6766). Patients in the early DOAC group had significantly lower odds of RIS than those in the late group (2.2% vs 2.9%, OR 0.72, 95% CI 0.52 to 0.98, p=0.04). No significant heterogeneity was observed among the trials reporting this composite outcome (I^2^=40%). See figure 2A for details. Furthermore, in the subgroup analysis for RIS, the pooled OR from eight observational studies was 0.76 (95% CI 0.50 to 1.14, p=0.18), with moderate heterogeneity (I^2^=53%). For two RCTs, the pooled OR was 0.61 (95% CI 0.38 to 0.98, p<0.05, I^2^=0%). The combined OR for RIS across all studies was 0.72 (95% CI 0.52 to 0.98, p<0.05, I^2^=40%). Please refer to figure 3A.

The outcome of intracerebral haemorrhage (ICH) was investigated in 11 studies (nine cohort studies and two RCTs),^89 17 18 21^,^25 28^ with 32 events (0.51%) in the early DOAC group (n=6250) and 63 events (0.93%) in the late DOAC group (n=6770). Patients who received early DOAC treatment had significantly lower odds of ICH (0.51% vs 0.93%, OR 0.45, 95% CI 0.29 to 0.70, p<0.05, I^2^=0%). Figure 2B provides further details. In the subgroup analysis of observational studies and RCTs for ICH, the pooled OR from nine cohort studies was 0.43 (95% CI 0.27 to 0.68, p<0.05), with no heterogeneity (I^2^=0%). In contrast, the pooled OR from two RCTs was 1.01 (95% CI 0.14 to 7.16, p=0.9). The combined OR for ICH across all studies was 0.45 (95% CI 0.29 to 0.70, p<0.05), with no heterogeneity (I^2^=0%). Moreover, the test for subgroup differences between observational studies and RCTs was not significant for either ICH (p=0.41) or RIS (p=0.50). Please refer to figure 3B.

Five studies^8 9 23 25 28^ reported rates of major haemorrhage (MH), 0.49% (early DOAC) and 0.75% (late DOAC), showing similar odds between the two groups (OR 0.69, 95% CI 0.37 to 1.31, p=0.26, I^2^=0%). Please refer to figures2C  3C for details. Two studies reported data on non-major haemorrhage (NMH).^8 20^ No statistically significant differences were noticed with the event rates for NMH (3.9% vs 3%, OR 1.5, 95% CI 0.8 to 2.8, p=0.21), with substantial heterogeneity (I^2^=63%). Please see figure 2D for further information. Subgroup analyses indicated that this heterogeneity affected both observational and RCT studies (refer to figure 3D). Furthermore, three studies provided data on systemic embolism,^8 23 25^ with no statistically significant differences between the two groups (OR 0.72, 95% CI 0.3 to 1.69, p=0.45), however, reported substantial heterogeneity (I^2^=63%) (refer to figure 2E). Moreover, all-cause mortality events were documented in four studies,^9 20 23 25^ showing no significant difference between the early and late DOAC groups (2.09% vs 2.46%, OR 0.81, 95% CI 0.38 to 1.73, p=0.09), with substantial heterogeneity (I^2^=54%) (refer to figure 2F). No significant subgroup differences were found among cohort and RCT studies on subgroup analysis, as shown by I² values of 0% (refer to figure 3E,F). While comorbidities, mean NIHSS, HAS-BLED and CHA2DS2-VASc scores were comparable across studies, the specific factors contributing to heterogeneity could not be identified.

In sensitivity analyses comparing DOAC initiation within 4 days versus after 4 days across cohort studies and RCTs, the early DOAC group showed a decreased risk of RIS (OR 0.64, 95% CI 0.42 to 0.99, I^2^=37%); however, no statistically significant difference was observed for ICH. The limited number of RCTs weakened the conclusion of the findings (online supplemental figure 1A,B). Furthermore, one opt-out sensitivity analysis was performed, which suggested the early DOAC group had reduced the RIS and ICH events. However, its risk of MH, NMH, systemic embolism and all-cause mortality varied across studies. There was some heterogeneity among studies likely due to differences in study design, populations and limited number of RCTs (online supplemental table 1).

## Discussion

This meta-analysis, comprising 11 studies (two RCTs and nine cohort studies), evaluated the efficacy and safety of early versus late DOAC initiation for secondary stroke prevention in patients with AF and AIS. The findings indicated that the early DOAC group had lower odds of RIS and ICH.

**Figure 4:**
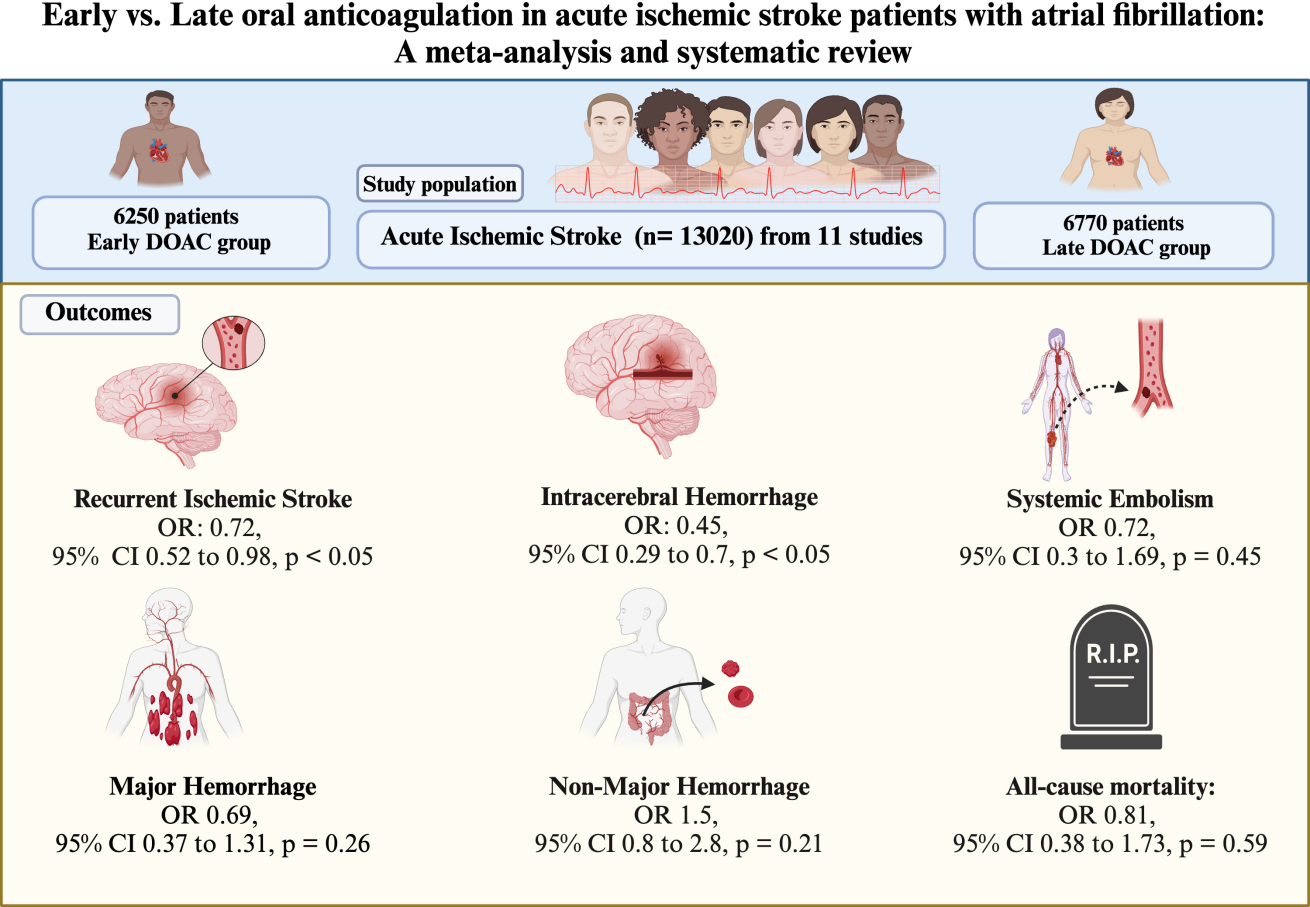
Central illustration. DOAC, direct oral anticoagulant.

Subgroup analyses among RCTs and cohort studies revealed that the early DOAC group had an overall statistically lower risk of RIS and ICH; however, the results were inconsistent between cohort studies and RCTs. Sensitivity analysis suggested that early DOAC (<4 days) decreased the risk of RIS without a statistically significant effect on ICH. Additionally, there was no statistically significant increase in the risk of MH, NMH, systemic embolism or all-cause mortality. However, the limited number of RCTs and substantial heterogeneity among cohort studies weaken the strength of these conclusions. Additional RCTs are needed to provide more definitive insights (figure 4). (online supplemental file 2)

ICH is the most gravid complication of DOAC after AIS and is associated with increased morbidity and mortality.^11^ As a result, a trend of delayed DOAC initiation was observed in earlier studies,^11 33 34^ particularly those conducted in the pre-DOAC era, which primarily used aspirin, heparin, low molecular weight heparin (LMWH)^4 10 16 19^ and VKAs.^12 26 27 29^ For instance, the RAF study demonstrated an underutilisation of DOACs (12.1%).^13^ Similarly, the ARISTOTLE study delayed apixaban initiation for at least 7 days after any AIS.^33^ In the ROCKET-AF trial, rivaroxaban was initiated at least 14 days after a minor AIS and 90 days after a major AIS.^34^ Various studies in the past have reported a higher incidence of ICH; however, they were from the pre-DOAC era and primarily used VKA and LMWH.^10 16 19 26 27 29^ However, DOAC has been established as the preferred alternative to VKA due to decreased haemorrhagic complications and faster onset of action.^21 35^

TIMING^9^ trial reported that the early treatment group (within 4 days) was non-inferior to the later treatment group (within 5–10 days) with the use of DOACs after AIS, with no ICH in any of the groups. Consistent with the recent studies,^8 9^ this meta-analysis findings were suggestive of the early DOAC being non-inferior to the later DOAC, with reduced odds of RIS, as reported among various studies.^8 17 20 21 23 25 26 28^ Furthermore, the early DOAC group showed a lower risk of symptomatic ICH compared with the late DOAC group.^8 9 12 18 20 21 24 25^ Additionally, in our meta-analysis, there were no statistically significant differences in adverse events such as MH, NMH and all-cause mortality among either group. DOAC has been shown to be a less frequent cause of MH; however, it is associated with NMH, such as gastrointestinal haemorrhage.^36^ This is an important cause of holding or withdrawing DOAC use in high-risk populations based on clinical relevance.^37^ In our study, only two studies reported the NMH; hence, the relevance could not be studied thoroughly. Future RCTs are needed to shed further light on this aspect.

A recent study, the ELAN trial,^8^ used DOAC and found the incidence of RIS to be 1.4% in the early treatment group versus 2.5% in the later treatment group (OR 0.57, 95% CI 0.29 to 1.07), while the incidence of ICH was comparable in both arms (0.2%). Additionally, the early DOAC group adopted a strategy of initiating DOAC within 48 hours following mild or moderate strokes and 6–7 days after major strokes, whereas the late DOAC cohort followed a strategy similar to EHRA-ESC guidelines,^14^ with DOAC initiated in 3–4 days after a minor stroke, 6–7 after a moderate stroke and 12–14 after a major stroke. These strategies differ from the 2021 AHA/ASA guideline,^15^ which recommends delaying initiation of DOAC beyond 14 days in high-risk patients to reduce the risk of ICH, whereas patients at low risk of ICH may initiate DOAC after 2–14 days to reduce the risk of RIS.

Differences in guideline recommendations stem from studies conducted in the pre-DOAC era^21^ and do not differentiate between the use of DOAC and VKA. In included studies, patients were stratified in early versus late DOAC groups based on the size of the infarct,^8 9 25 26^ NIHSS score^9 18^ and individual risk factors (HAS-BLED, CHADSVASc score).^10 23^

The safety and efficacy of DOACs are well documented in patients with low-risk profiles, such as those with small infarct size, low CHADSVASc scores and low HAS-BLED scores.^8 9^ Further studies are required, especially in high-risk populations, such as those with large infarct sizes and high NIHSS and HAS-BLED scores, a recent history of reperfusion treatment and ischaemic stroke while on DOAC treatment ‘DOAC failure’. Such an investigation would provide confidence in the generalisability of results. Ongoing clinical trials will provide further insights: OPTIMAS (EudraCT, 2018-003859-38; UK), START (NCT03021928; USA) and ASAP (NCT06057467; China) to address this knowledge gap.

Jiang *et al* conducted a previous meta-analysis on this topic, comparing early versus late initiation of DOAC. Their findings aligned with our study, showing a decreased risk of RIS in the early group. However, they did not find statistically significant differences in the risk of ICH.^38^ Additionally, their analysis included studies that employed various OAC strategies such as DOAC, VKA and LMWH. Based on recent literature, DOACs have been the preferred OAC choice due to their similar therapeutic effects to VKA and reduced bleeding risks.^21 35 39^ ^40^ By combining data from 11 studies that primarily used DOACs, our analysis supports the trend of early DOAC initiation for secondary stroke prophylaxis in patients with AIS and AF; however, further RCTs are needed to provide further insights.

## Limitations

Our meta-analysis has several limitations. (1) Studies categorised early and late DOAC groups solely by initiation timing, without considering stroke severity, NIHSS or HAS-BLED scores. (2) The mean NIHSS scores were 6.6 for the early group and 8.4 for the late group, with a mean HAS-BLED score of 4.3 in both, reflecting a higher disease burden in the late group; we could not analyse these differences. (3) The majority of included studies were observational and did not adequately address confounding factors, leading to potential selection bias. (4) We could not comprehensively determine the timing of early DOAC administration due to variability in definitions among studies. (5) Significant heterogeneity among subgroups and the limited number of RCTs further weakened our conclusions.

## Conclusion

This meta-analysis of 11 studies (two RCTs and nine cohort studies) investigated the trend of optimal timing for initiating direct oral anticoagulants (DOACs) for secondary stroke prevention in patients with AF and AIS. The results indicate that a trend of early DOAC initiation is associated with lower rates of RIS and ICH compared with later initiation. However, no significant differences were observed in MH, NMH, systemic embolism or all-cause mortality. The conclusions are weakened by the limited number of RCTs and considerable heterogeneity among studies, underscoring the need for additional research to provide more definitive insights.

## supplementary material

10.1136/openhrt-2024-003002 online supplemental file 1

10.1136/openhrt-2024-003002 online supplemental file 2

10.1136/openhrt-2024-003002 online supplemental file 3

10.1136/openhrt-2024-003002 online supplemental file 4

10.1136/openhrt-2024-003002 online supplemental file 5

## Data Availability

Data are available in a public, open access repository.
