# Supplementary material for: Optimal timing of oral anticoagulation initiation in patients with acute ischaemic stroke and atrial fibrillation: a comprehensive meta-analysis and systematic review
Source: Open Heart. 2024 Nov 27;11(2):e003002. doi: 10.1136/openhrt-2024-003002 (PMC11603680; doi:10.1136/openhrt-2024-003002)
Supplement: online supplemental file 1 [file openhrt-11-2-s001.pdf]

Data sharing statement

The data that support the findings of this study are openly available.

1. Fischer U, Koga M, Strbian D, Branca M, Abend S, Sven Trelle, et al. Early versus later anticoagulation for stroke with atrial fibrillation. The New England Journal of Medicine 2023;388. https://doi.org/10.1056/nejmoa2303048. (Open Access)
2. Oldgren J, Åsberg S, Hijazi Z, Wester P, Bertilsson M, Norrving B. Early Versus Delayed Non–Vitamin K Antagonist Oral Anticoagulant Therapy After Acute Ischemic Stroke in Atrial Fibrillation (TIMING): A Registry-Based Randomized Controlled Noninferiority Study. Circulation 2022;146:1056–66. https://doi.org/10.1161/circulationaha.122.060666. (Open Access)
3. Cappellari M, Carletti M, Danese A, Bovi P. Early introduction of direct oral anticoagulants in cardioembolic stroke patients with non-valvular atrial fibrillation. Journal of Thrombosis and Thrombolysis. https://doi.org/10.1007/s11239-016-1393-9 (Springer Nature has partnered with Copyright Clearance Center's RightsLink service to offer a variety of options for reusing this content).
4. De M, Seiffge DJ, Schaedelin S, Wilson D, Caso V, Acciarresi M, et al. Early versus late start of direct oral anticoagulants after acute ischaemic stroke linked to atrial fibrillation: an observational study and individual patient data pooled analysis. Journal of Neurology, Neurosurgery, and Psychiatry 2021;93:119–25. https://doi.org/10.1136/jnnp-2021-327236

   (BMJ Publishing Group Ltd. has partnered with Copyright Clearance Center's RightsLink service to offer a variety of options for reusing this content.)
5. Wilson D, Ambler G, Banerjee G, Shakeshaft C, Cohen H, Yousry TA, et al. Early versus

late anticoagulation for ischaemic stroke associated with atrial fibrillation: multicentre cohort study. Journal of Neurology, Neurosurgery & Psychiatry 2018;90:320–5. https://doi.org/10.1136/jnnp-2018-318890. (Open Access)

6. Sharobeam A, Lin L, Lam C, *et al* Early anticoagulation in patients with stroke and atrial fibrillation is associated with fewer ischaemic lesions at 1 month: the ATTUNE study *Stroke and Vascular Neurology* 2024;**9** https://doi.org/10.1136/svn-2023-002357. (Open Access)
7. Maurizio Paciaroni, Agnelli G, Michela Giustozzi, Georgios Tsivgoulis, Yaghi S, Brian Mac Grory, et al. Timing of initiation of oral anticoagulants in patients with acute ischemic stroke and atrial fibrillation comparing posterior and anterior circulation strokes. European Stroke Journal 2020;5:374–83. https://doi.org/10.1177/2396987320937116. (Open Access)
8. Mizoguchi T, Tanaka K, Toyoda K, Yoshimura S, Itabashi R, Takagi M, et al. Early Initiation of Direct Oral Anticoagulants After Onset of Stroke and Short- and Long-Term Outcomes of Patients With Nonvalvular Atrial Fibrillation. Stroke 2020;51:883–91. https://doi.org/10.1161/STROKEAHA.119.028118. (Open Access)
9. Macha K, Volbers B, Bobinger T, Kurka N, Breuer L, Huttner HB, et al. Early Initiation of Anticoagulation with Direct Oral Anticoagulants in Patients after Transient Ischemic Attack or Ischemic Stroke. Journal of Stroke and Cerebrovascular Diseases 2016;25:2317–21. https://doi.org/10.1016/j.jstrokecerebrovasdis.2016.06.031 (Elsevier has partnered with Copyright Clearance Center's RightsLink service to offer a variety of options for reusing this content).

10. Kimura S, Toyoda K, Yoshimura S, Minematsu K, Yasaka M, Paciaroni M, et al. Practical “1-2-3-4-Day” Rule for Starting Direct Oral Anticoagulants After Ischemic Stroke With Atrial Fibrillation: Combined Hospital-Based Cohort Study. Stroke 2022;53:1540–1549. https://doi.org/10.1161/STROKEAHA.121.036695. (Open Access)
11. Yasaka M, Minematsu K, Toyoda K, Mori E, Hirano T, Hamasaki T, et al. (2019) Rivaroxaban administration after acute ischemic stroke: The RELAXED study. PLoS ONE 14(2): e0212354. https://doi.org/10.1371/journal.pone.0212354. (Open Access)
