# Supplementary material for: Optimal timing of oral anticoagulation initiation in patients with acute ischaemic stroke and atrial fibrillation: a comprehensive meta-analysis and systematic review
Source: Open Heart. 2024 Nov 27;11(2):e003002. doi: 10.1136/openhrt-2024-003002 (PMC11603680; doi:10.1136/openhrt-2024-003002)

Supplementary Figure 1: Sensitivity Analysis among Early (< 4 days) and Late (> 4 days) groups.

A. **Recurrent Ischemic Stroke**

Study or Subgroup	Early AC Events	Early AC Total	Late AC Events	Late AC Total	Weight	Odds Ratio M-H, Random, 95% CI	Year
**1.1.1 Observational**							
Cappellari et al., 2016	0	97	0	50		Not estimable	2016
Wilson et al., 2018	3	358	16	997	9.6%	0.52 [0.15, 1.79]	2018
Yasaka et al., 2019	9	584	21	721	18.0%	0.52 [0.24, 1.15]	2019
Mizoguchi et al., 2020	11	223	6	276	13.0%	2.33 [0.85, 6.42]	2020
Kimura et al., 2022	13	785	32	1012	22.3%	0.52 [0.27, 0.99]	2022
Sharobeam et al., 2023	9	107	18	101	16.4%	0.42 [0.18, 0.99]	2023
**Subtotal (95% CI)**		**2154**		**3157**	**79.2%**	**0.64 [0.37, 1.12]**	
Total events	45		93				
Heterogeneity: $Tau^2 = 0.19$; $Chi^2 = 7.95$, df = 4 (P = 0.09); $I^2 = 50\%$							
Test for overall effect: Z = 1.56 (P = 0.12)							
**1.1.2 RCT**							
Oldgreen et al., 2022	14	450	20	438	20.8%	0.67 [0.33, 1.35]	2022
**Subtotal (95% CI)**		**450**		**438**	**20.8%**	**0.67 [0.33, 1.35]**	
Total events	14		20				
Heterogeneity: Not applicable							
Test for overall effect: Z = 1.12 (P = 0.26)							
**Total (95% CI)**		**2604**		**3595**	**100.0%**	**0.64 [0.42, 0.99]**	
Total events	59		113				
Heterogeneity: $Tau^2 = 0.11$; $Chi^2 = 8.00$, df = 5 (P = 0.16); $I^2 = 37\%$							
Test for overall effect: Z = 2.00 (P = 0.05)							
Test for subgroup differences: $Chi^2 = 0.01$, df = 1 (P = 0.93), $I^2 = 0\%$							

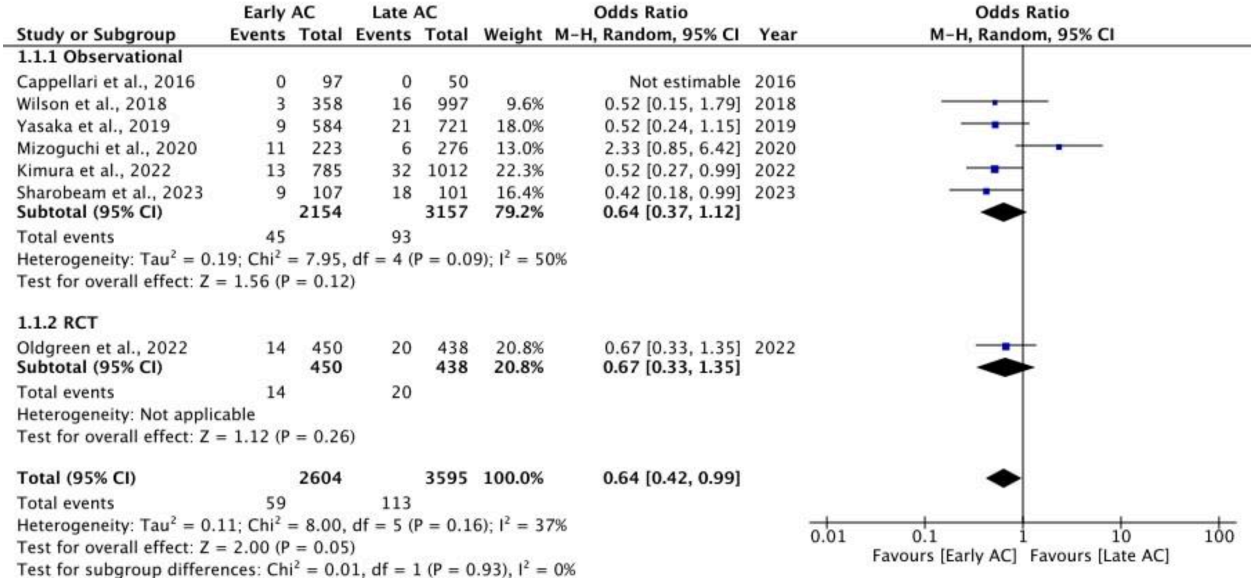

B. **Intracerebral Hemorrhage.**

Study or Subgroup	Early AC Events	Early AC Total	Late AC Events	Late AC Total	Weight	Odds Ratio M-H, Random, 95% CI	Year
**1.2.1 Observational**							
Cappellari et al., 2016	6	97	2	50	30.1%	1.58 [0.31, 8.14]	2016
Wilson et al., 2018	0	358	2	997	8.7%	0.56 [0.03, 11.60]	2018
Yasaka et al., 2019	1	584	4	721	16.8%	0.31 [0.03, 2.76]	2019
Mizoguchi et al., 2020	0	223	2	276	8.7%	0.25 [0.01, 5.14]	2020
Kimura et al., 2022	2	785	4	1012	27.9%	0.64 [0.12, 3.52]	2022
Sharobeam et al., 2023	0	107	1	101	7.8%	0.31 [0.01, 7.74]	2023
**Subtotal (95% CI)**		**2154**		**3157**	**100.0%**	**0.64 [0.26, 1.57]**	
Total events	9		15				
Heterogeneity: $Tau^2 = 0.00$; $Chi^2 = 2.19$, df = 5 (P = 0.82); $I^2 = 0\%$							
Test for overall effect: Z = 0.98 (P = 0.33)							
**1.2.2 RCT**							
Oldgreen et al., 2022	0	450	0	438		Not estimable	2022
**Subtotal (95% CI)**		**450**		**438**		**Not estimable**	
Total events	0		0				
Heterogeneity: Not applicable							
Test for overall effect: Not applicable							
**Total (95% CI)**		**2604**		**3595**	**100.0%**	**0.64 [0.26, 1.57]**	
Total events	9		15				
Heterogeneity: $Tau^2 = 0.00$; $Chi^2 = 2.19$, df = 5 (P = 0.82); $I^2 = 0\%$							
Test for overall effect: Z = 0.98 (P = 0.33)							
Test for subgroup differences: Not applicable							

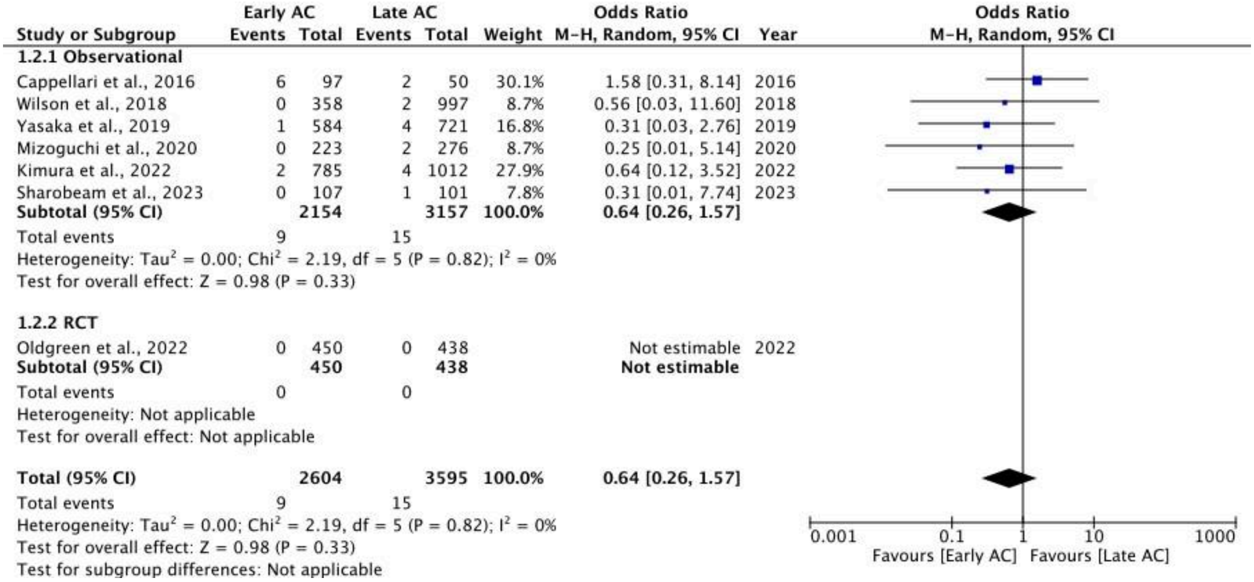

Supplement: online supplemental file 3 [file openhrt-11-2-s003.pdf]
