# Supplementary material for: Optimal timing of oral anticoagulation initiation in patients with acute ischaemic stroke and atrial fibrillation: a comprehensive meta-analysis and systematic review
Source: Open Heart. 2024 Nov 27;11(2):e003002. doi: 10.1136/openhrt-2024-003002 (PMC11603680; doi:10.1136/openhrt-2024-003002)

Supplementary Figure 2: Risk of Bias Summary of RCTs.

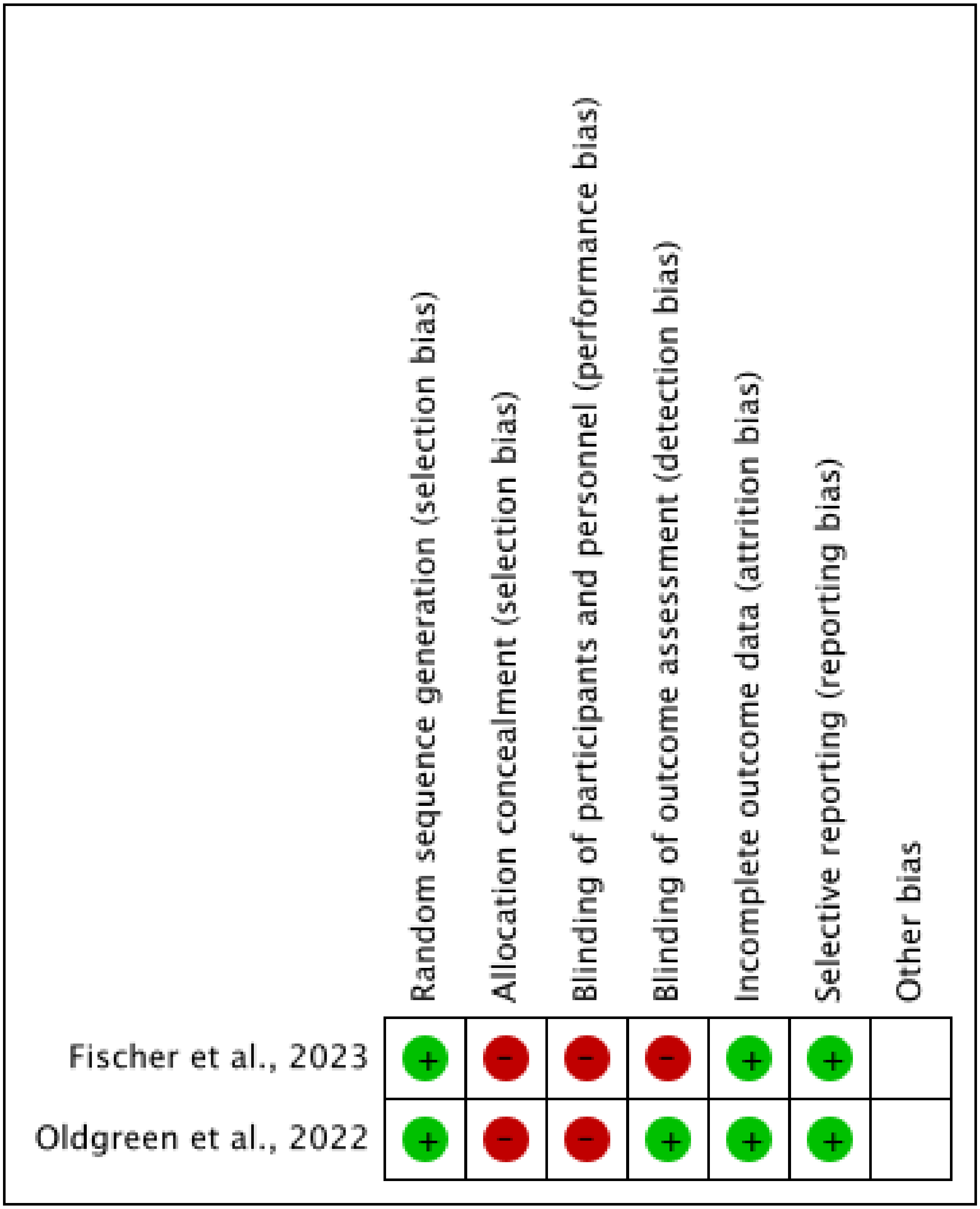

Supplement: online supplemental file 4 [file openhrt-11-2-s004.pdf]
