# Supplementary material for: Optimal timing of oral anticoagulation initiation in patients with acute ischaemic stroke and atrial fibrillation: a comprehensive meta-analysis and systematic review
Source: Open Heart. 2024 Nov 27;11(2):e003002. doi: 10.1136/openhrt-2024-003002 (PMC11603680; doi:10.1136/openhrt-2024-003002)
Supplement: online supplemental file 5 [file openhrt-11-2-s005.pdf]

Supplementary Table 1: One opt-out sensitivity analysis.

Study	RIS	ICH	Major Haemorrhage	Embolism	All mortality
Final outcome	0.72 [0.52, 0.98]	0.45 [0.29, 0.70]	0.69 [0.37, 1.31]	0.72 [0.30, 1.69]	0.81 [0.38, 1.73]
Study excluded					
Marchis et al., 2022	0.65 [0.49, 0.86]	0.45 [0.29, 0.71]			
Fischer et al., 2023	0.74 [0.52, 1.06]	0.43 [0.27, 0.68]	0.72 [0.35, 1.47]	0.88 [0.25, 3.17]	
Kimura et al., 2022	0.75 [0.53, 1.06]	0.44 [0.28, 0.69]	0.65 [0.29, 1.47]	0.93 [0.24, 3.67]	0.61 [0.18, 2.02]
Macha et al., 2016		0.47 [0.30, 0.73]			
Oldgreen et al., 2022	0.72 [0.51, 1.03]	0.45 [0.29, 0.70]	0.69 [0.37, 1.31]		0.67 [0.17, 2.72]
Sharobeam et al., 2023	0.76 [0.55, 1.05]	0.45 [0.29, 0.71]			

Wilson et al., 2018	0.73 [0.52, 1.02]	0.45 [0.29, 0.70]			1.11 [0.70, 1.78]
Pachiaroni et al., 2020	0.71 [0.49, 1.04]	0.57 [0.28, 1.16]			
Mizoguchi et al., 2020	0.67 [0.52, 0.86]	0.46 [0.29, 0.71]	0.71 [0.36, 1.41]	0.48 [0.28, 0.82]	0.74 [0.30, 1.82]
Cappellari et al., 2016	0.72 [0.52, 0.98]	0.41 [0.26, 0.64]			
Yasaka et al., 2019	0.74 [0.53, 1.05]	0.46 [0.29, 0.72]	0.69 [0.33, 1.46]		
